# Galeazzi Fracture Dislocations: An Illustrated Review

**DOI:** 10.7759/cureus.9367

**Published:** 2020-07-24

**Authors:** Turki Alajmi

**Affiliations:** 1 Orthopedics and Traumatology, Prince Mohammed Bin Abdulaziz Hospital, Riyadh, SAU

**Keywords:** radius fracture, distal radioulnar joint, fracture dislocation, galeazzi, tfcc

## Abstract

Galeazzi fracture dislocations are a fracture of the distal one third of the radius shaft with a concomitant dislocation of the distal radioulnar joint (DRUJ). These injuries usually occur by axial loading on an outstretched arm with pronation or supination of the wrist which determines the angulation of the fracture. Surgical treatment has been historically by the anterior (volar) approach to the forearm with plate fixation with or without pinning of the distal radioulnar joint. Failed or inadequate treatment may lead to complications including chronic pain, malunion or instability of the DRUJ that may warrant salvage procedures.

## Introduction and background

Galeazzi fracture dislocations are a fracture of the distal one third of the ulna’s shaft with a concomitant dislocation of the distal radioulnar joint (DRUJ). First it was described by the British surgeon Sir Astley Cooper in 1822 and then it was named after Galeazzi who first reported a series of 18 cases in 1934 in which he described the mechanism, incidence, and management of this injury [1]. They represent approximately 7% of adult and 3% of pediatric forearm fractures, with closed reduction and casting being the gold standard of treatment in pediatrics and open reduction and internal fixation in adults in order to anatomically restore the radial bow to avoid any functional deficit [2]. This fracture has been notoriously known for its instability, and delayed or inadequate treatment may result in dreadful complications that could have a substantial effect on the outcomes of the fracture [3].

## Review

Pathoanatomy

**Figure 1:**
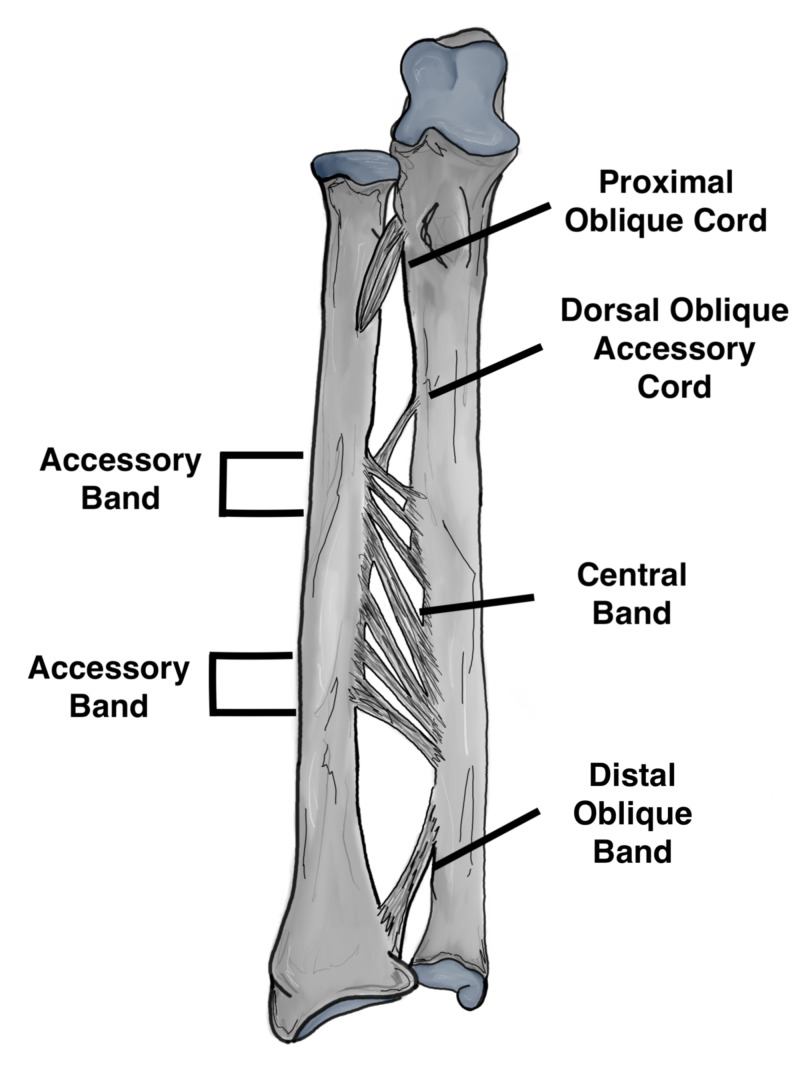
Components of the interosseous membrane Original illustration by Alswaji G.F.

**Figure 2:**
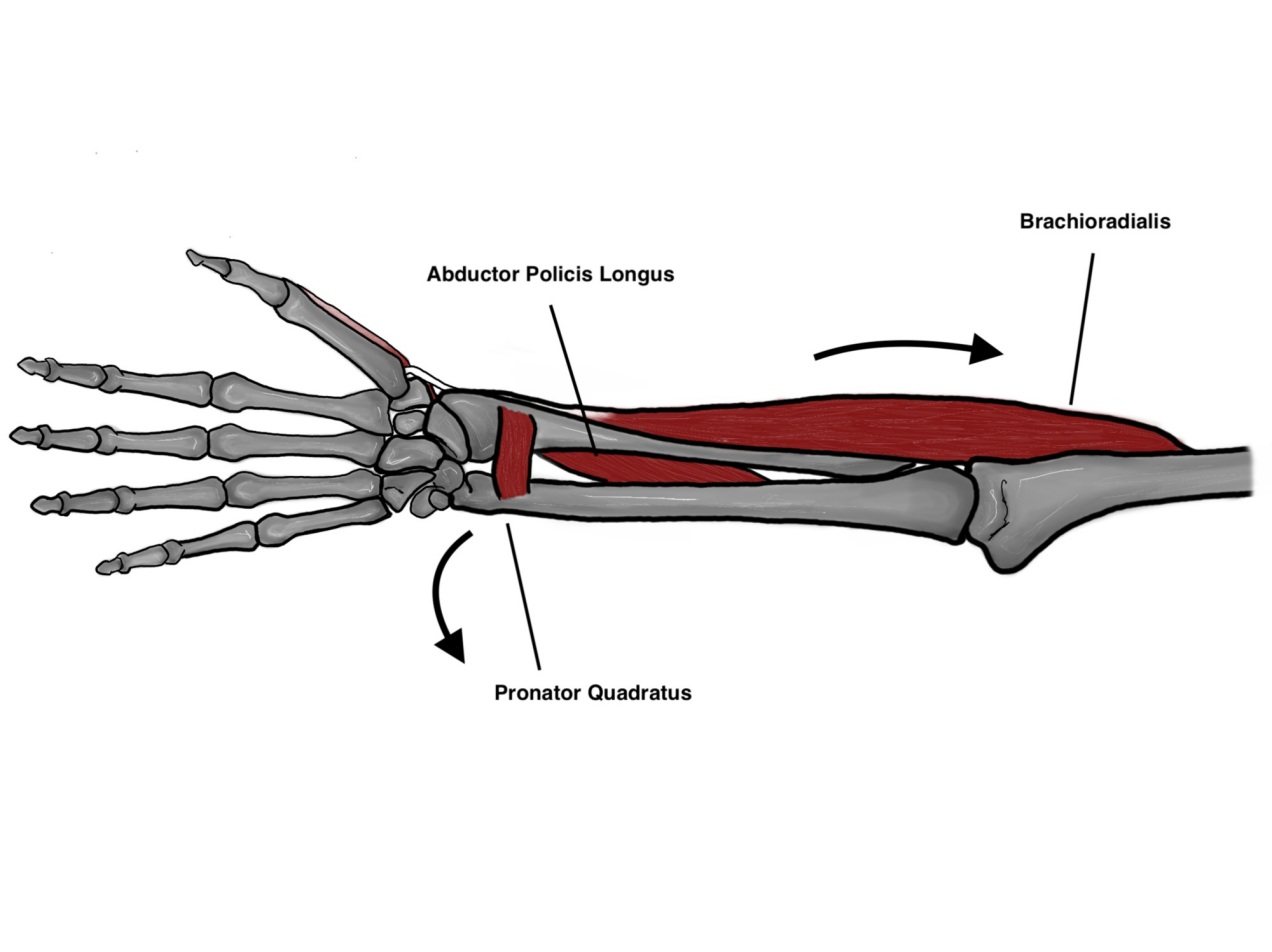
Deforming forces acting on the distal fragment Original illustration by Alswaji G.F.

**Figure 3:**
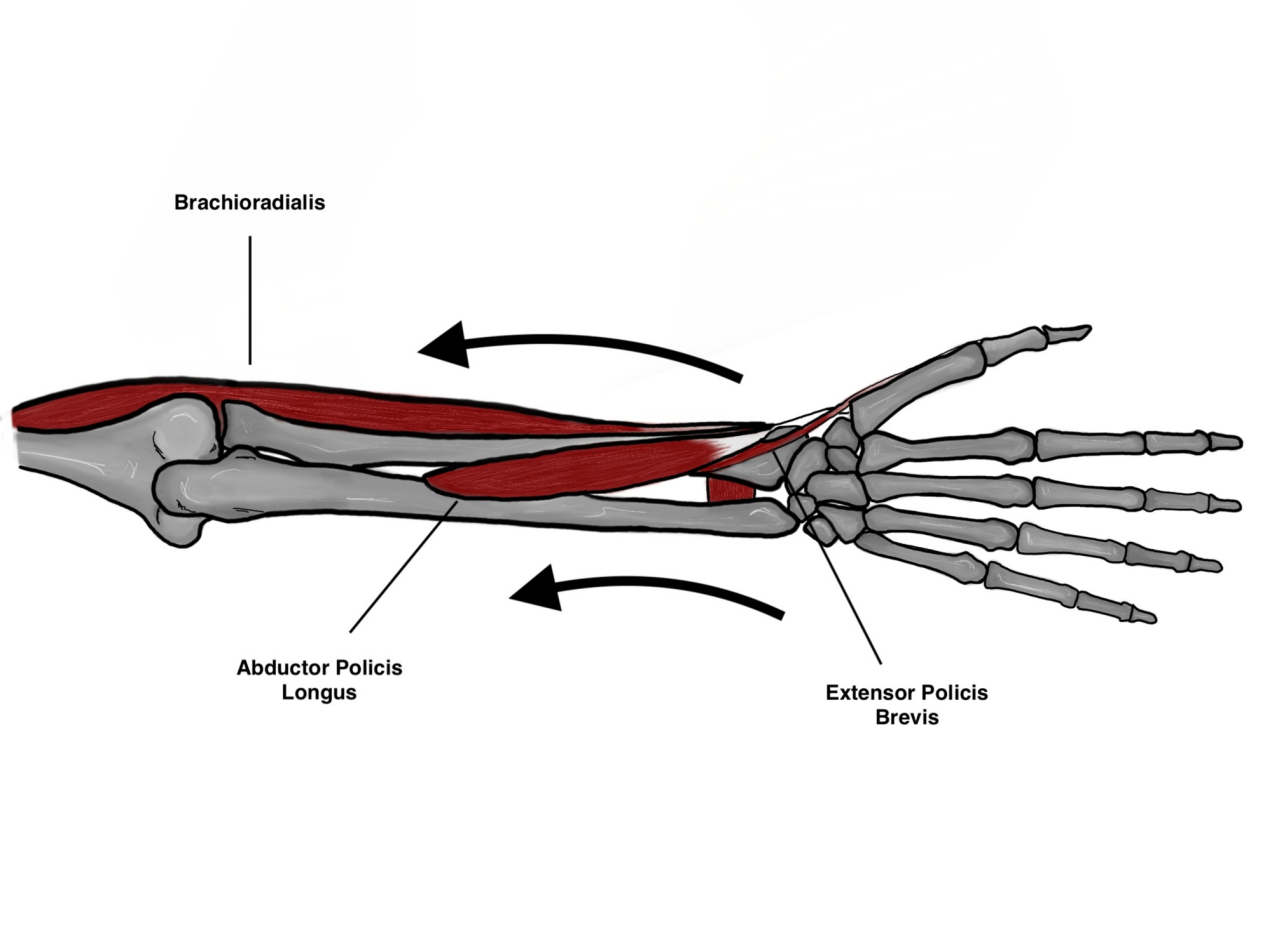
Deforming forces acting on the distal fragment Original illustration by Alswaji G.F.

**Figure 4:**
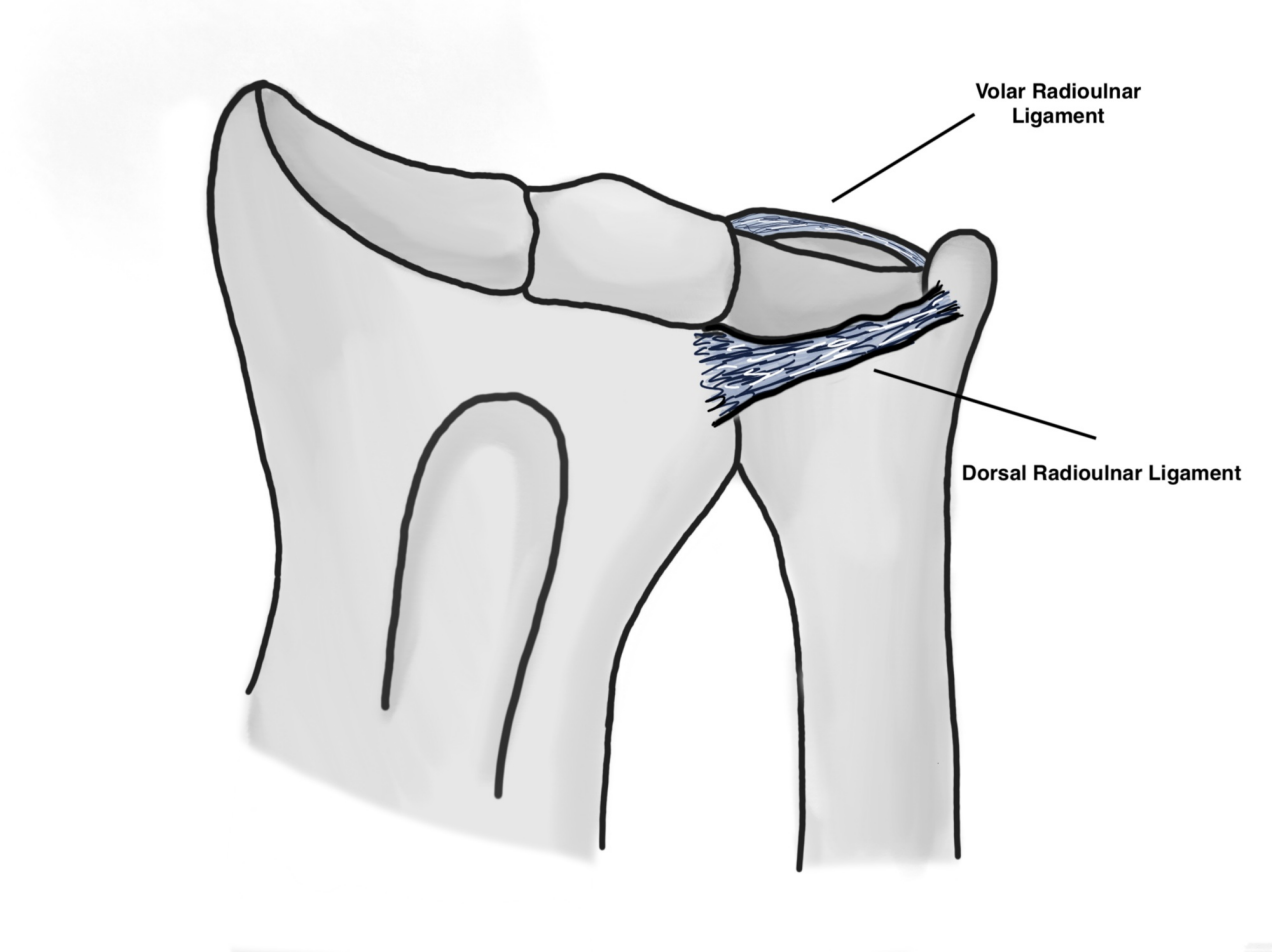
Distal radioulnar ligaments Original illustration by Alswaji G.F.

The radius and ulna are held together by the interosseous membrane (IOM) which is composed of the following: the proximal cords, accessory bands, distal band and finally the central band which is the strongest component of the IOM (Figure 1). The IOM is a relatively weak attachment to the distal one third of the radius which may predispose it to subsequent shortening if an injury occurs through it. The IOM has the following functions that are of biomechanical importance: 1) Load transfer from the radius to the ulna, 2) Load transfer from wrist joint to the elbow, 3) Maintains a stable DRUJ, 4) Maintains forearm stability throughout range of motion [4]. A number of deforming muscular forces are exerted on the distal radius. The abductor pollicis longus and extensor pollicis brevis exert a shortening force upon the distal radius, the pronator quadratus muscle also exerts a rotational force, and finally the brachioradialis pulls the distal radius fragment proximally (Figures 2, 3) [5]. Furthermore, the main stabilizer of the distal radioulnar joint is the triangular fibrocartilage complex (TFCC) which originates between the sigmoid notch and the lunate fossa on the radius, and inserts on the ulnar styloid and the fovea [6]. The dorsal and volar radioulnar ligaments of the TFCC also are paramount to maintain stability of the ulna and they are the main stabilizers of the DRUJ within the TFCC (Figure 4) [6].

Classification

**Figure 5:**
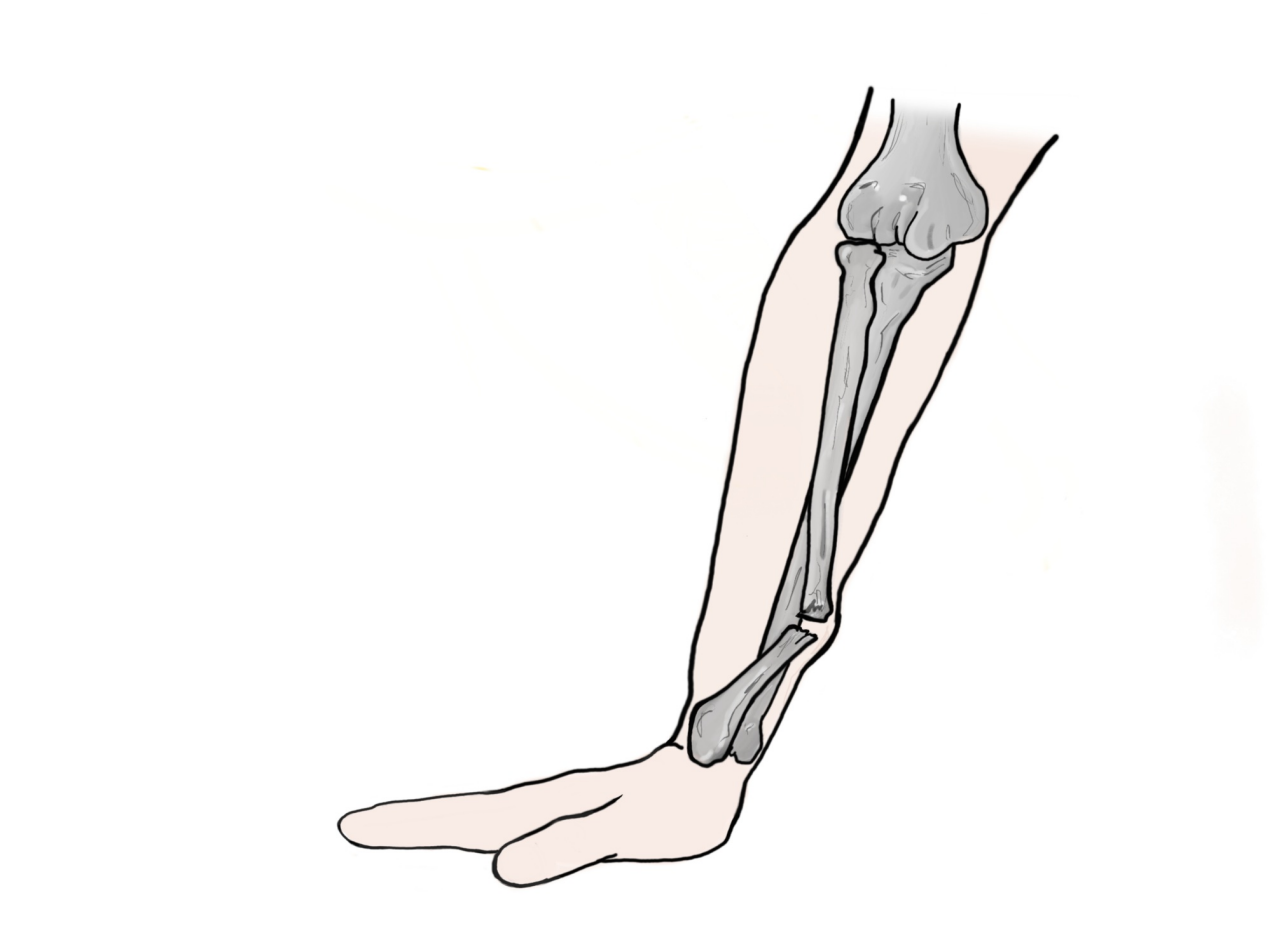
Apex volar galeazzi fracture Original illustration by Alswaji G.F.

**Figure 6:**
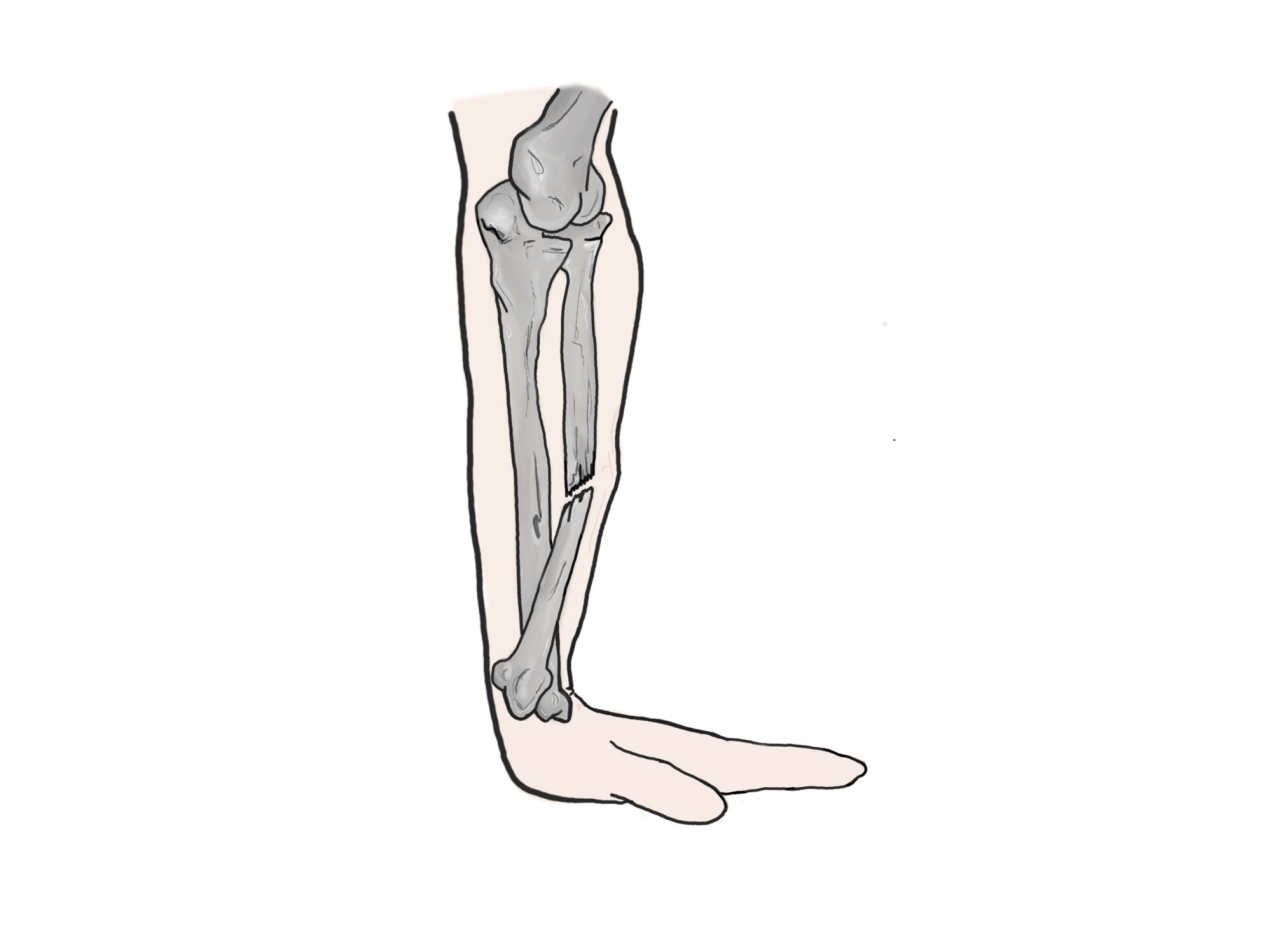
Apex dorsal galeazzi fracture Original illustration by Alswaji G.F.

There have been several classification systems to classify Galeazzi fracture dislocation, the first was described by Walsh et al. in a report of 41 pediatric fractures which included: Type 1 which is characterized by a dorsal displacement of the distal radius (apex volar), and it is caused by axial load applied to the forearm while the forearm is in supination (Figure 5) [7]. Type 2 which is characterized by volar (posterior) displacement of the distal radius which makes it an apex dorsal (Figure 6). A second classification system was proposed by Rettig and Raskin in 2001 which classifies the fracture based on the distance from the distal radioulnar joint, >7.5 cm or <7.5 cm [8]. Twenty-two of the 40 fractures had radius fractures <7.5 cm from the articular surface and 12 of these fractures showed intraoperative instability of the DRUJ. On the other hand, the remainder 18 fractures were >7.5 cm from the articular surface and only one of them was shown to intraoperative instability of the DRUJ. Beneyto et al. also classified Galeazzi fractures into three types based on the location of the distal radius fracture; type I was 0-10 cm from the tip of radial styloid, type II was 10-15 cm, and type III was >15 cm away from the radial styloid [9]. The worse results were noted in patients with type I fractures.

Diagnosis

**Figure 7:**
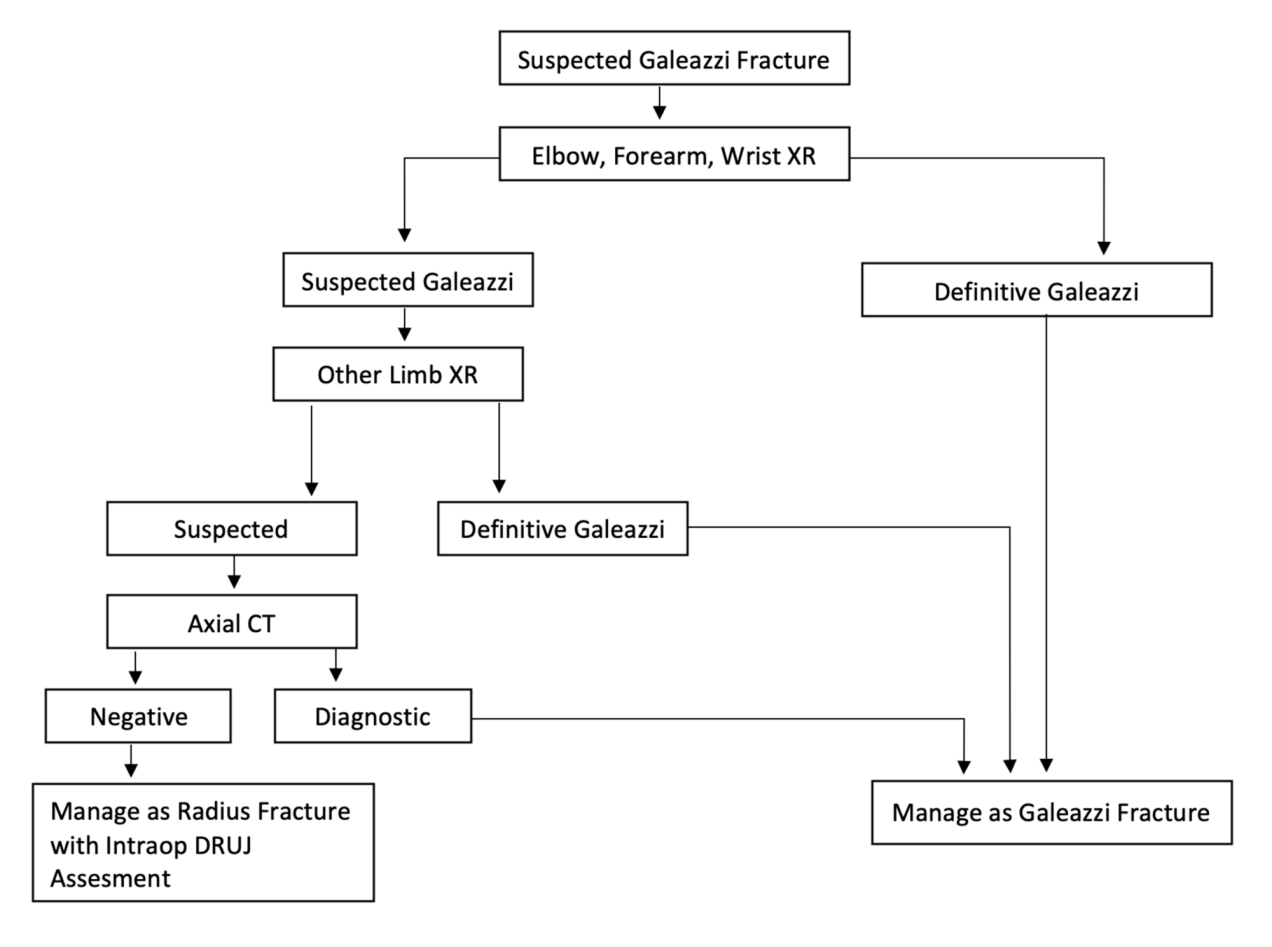
Diagnostic algorithm Proposed by the author.

A systemic approach should be utilized with any patient presenting with any orthopedic injury. In cases of an open fracture or high energy injury the Advanced Trauma Life Support protocol should be initiated to rule out any life-threatening injuries or hemorrhage. In cases of a low energy isolated Galeazzi fracture clinical examination usually reveals gross deformity and/or swelling on inspection. Tenderness to palpitation will be obvious in the site of the distal radius fracture and the distal radioulnar joint. Although painful, gentle passive and active wrist flexion and extension along with forearm rotation can be attempted. Prominent ulnar head either dorsally or volary along with distal radioulnar tenderness is characteristic for DRUJ injury [3]. Shortening of the radius may be evident depending on the extent and severity of the injury. A comprehensive neurovascular examination is mandatory although neurovascular injuries in Galeazzi fractures are rare [10]. Radiographic assessment should include dedicated X-rays of the wrist, forearm and elbow. The finding of the radius fracture and disruption of the DRUJ confirms the diagnosis of Galeazzi fracture dislocation. Contralateral wrist X-rays for comparison may aid in the diagnosis. Findings suggestive of DRUJ injury on plain radiographs include: Radius shortening >5 mm relative to the ulna, fracture of the base of the ulnar styloid, asymmetry compared to the contralateral uninjured limb radiographs, widening of the DRUJ on anteroposterior radiographs, and on lateral radiographs subluxation or dislocation of the radius relative to the ulna [11]. In the setting of negative radiographs but a high index of suspicion for DRUJ injury, axial CT has been recommended [12]. Currently the use of MRI in the diagnosis of Galeazzi fractures has not been clearly established [13]. Figure 7 shows a proposed diagnostic algorithm by the author.

Management

**Figure 8:**
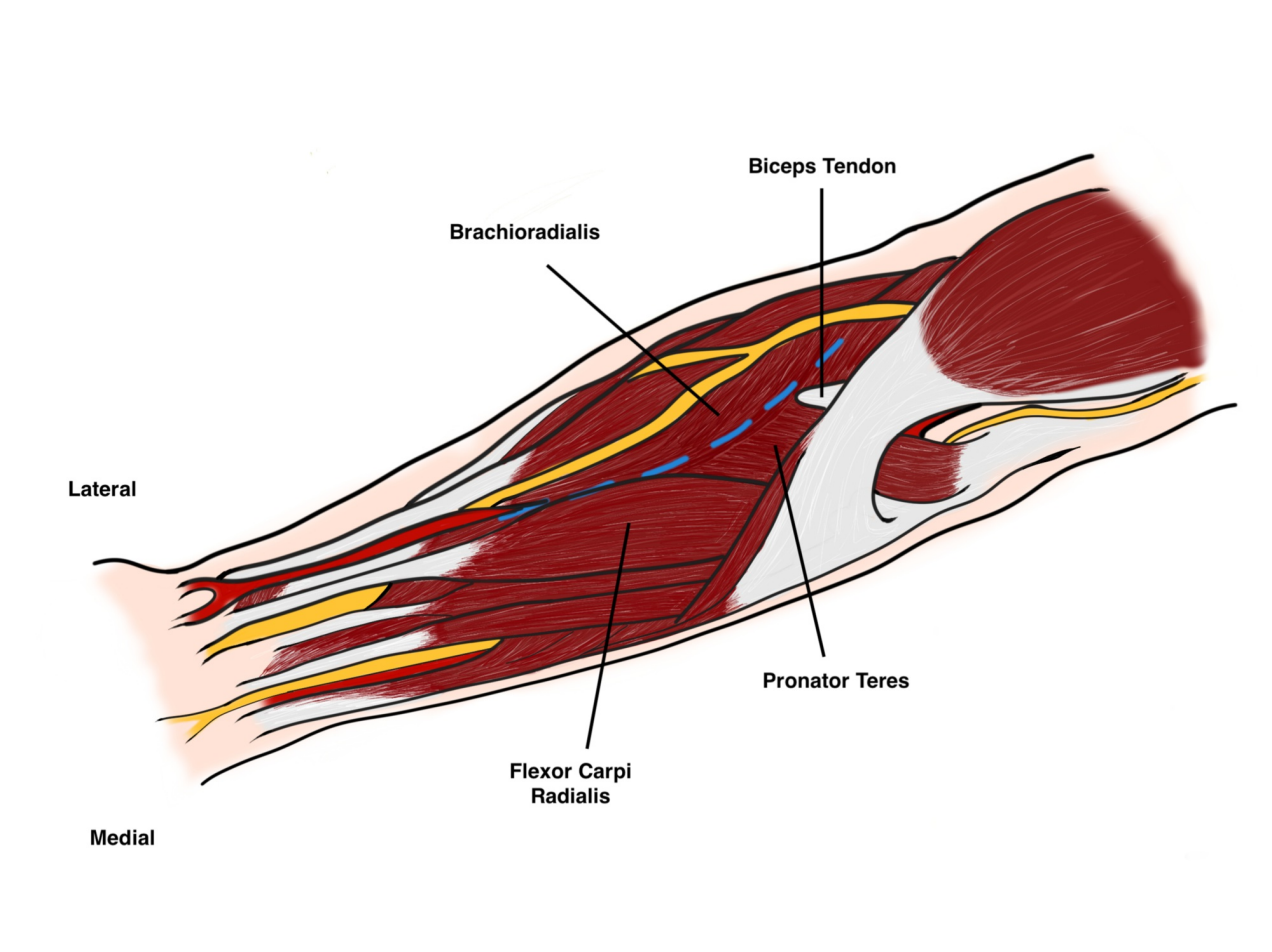
Anterior volar incision (Henry's Approach) Original illustration by Alswaji G.F.

In adults, Galeazzi fractures are known to be the “Fracture of Necessity”, necessitating open reduction and internal fixation to achieve satisfactory outcomes. This is largely due to the inherent instability of Galeazzi fractures and the deforming forces that were mentioned previously [14]. If treated conservatively in adults, the majority of Galeazzi fracture patient will almost always achieve non-satisfactory outcomes [15]. The Volar (Henry [16]) approach is classically utilized to access fractures of the middle and distal thirds of the radial shaft [17]. Although some surgeons may differ or disagree, described here is the author’s preferred method which is most commonly utilized. Prior to the incision and tourniquet inflation, it is best to avoid exsanguinating the limb in order to easily identify the radial artery by the two venae comitantes accompanying it. The landmarks are the radial head or lateral to the biceps tendon proximally to the radial styloid distally, the incision should be centered on the fracture site and can either be straight or curved proximally while the forearm is supinated (Figure 8).

**Figure 9:**
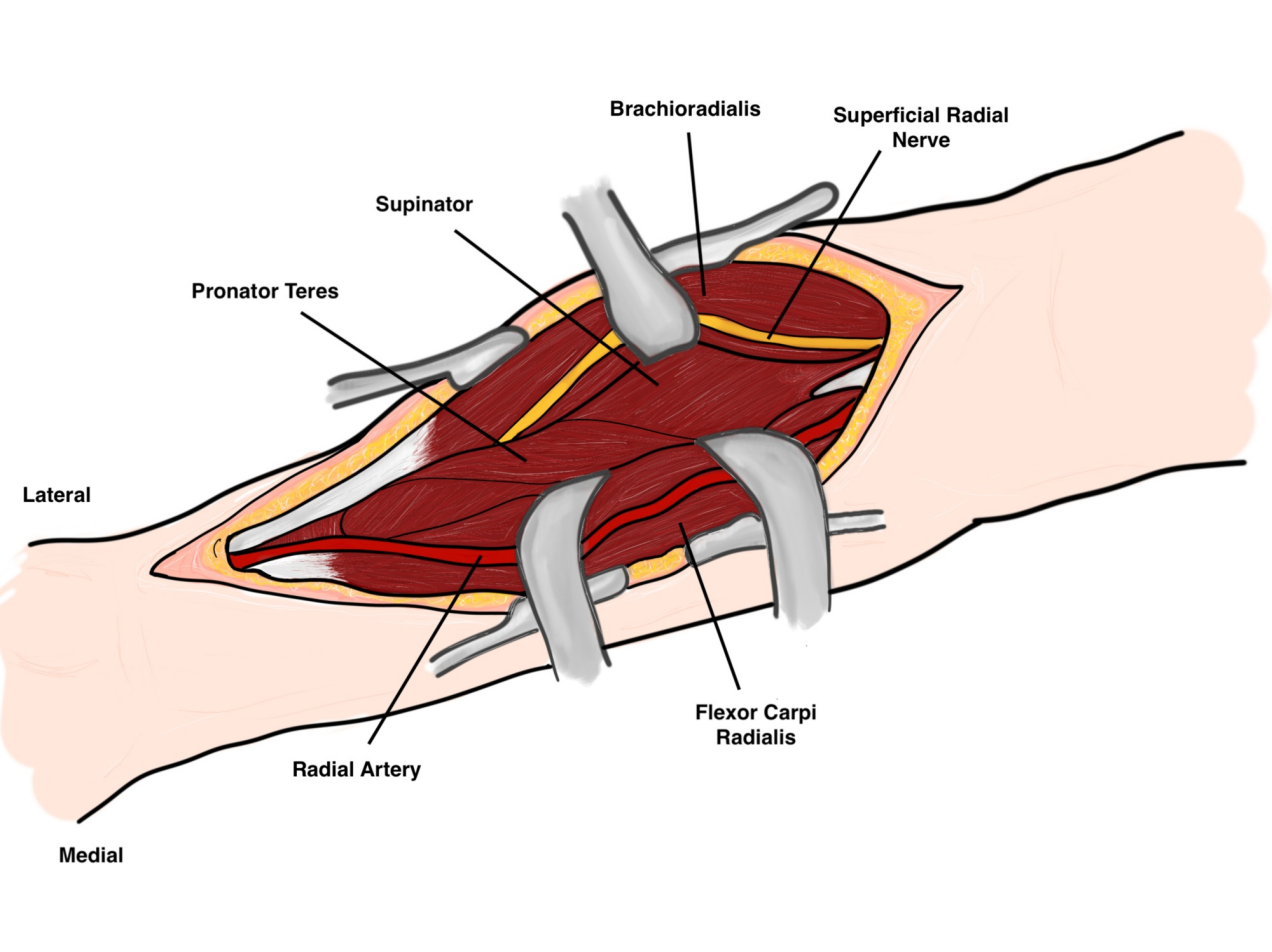
Superficial dissection Original illustration by Alswaji G.F.

After dissection of the subcutaneous fat, the proximal intermuscular interval is between the pronator teres and brachioradialis, while distally it is between the brachioradialis and the flexor carpi radialis (FCR). Careful incision of the fascia between the brachioradialis and the FCR as the radial artery lies directly below the medial edge of the brachioradialis midway in the forearm. The radial artery should be identified, protected, and freed along its length to allow it to be retracted medially. Under the brachioradialis muscle belly lies the superficial radial nerve which should also be identified and protected as injury or damage may cause neuromas (Figure 9).

**Figure 10:**
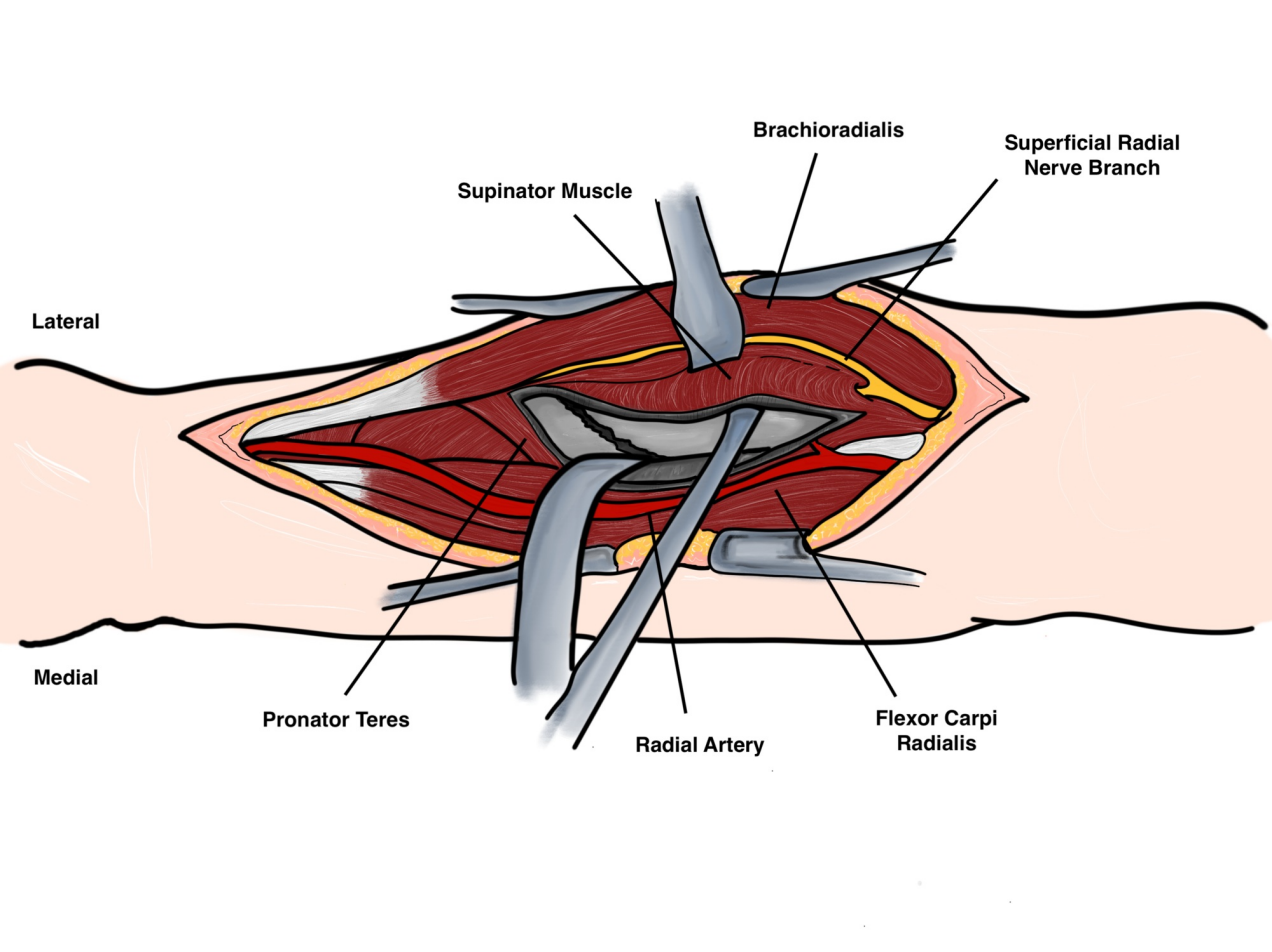
Deep dissection Original illustration by Alswaji G.F.

Deep dissection varies depending on the location. Proximally the posterior interosseous nerve (PIN) is at risk. In order to expose the proximal radius safely the forearm should be supinated in order to move the PIN laterally away from the surgical field. With this maneuver the insertion of the supinator muscle on the anterior radius is exposed and it is incised laterally and retracted with caution to avoid injury to the PIN (Figure 10).

**Figure 11:**
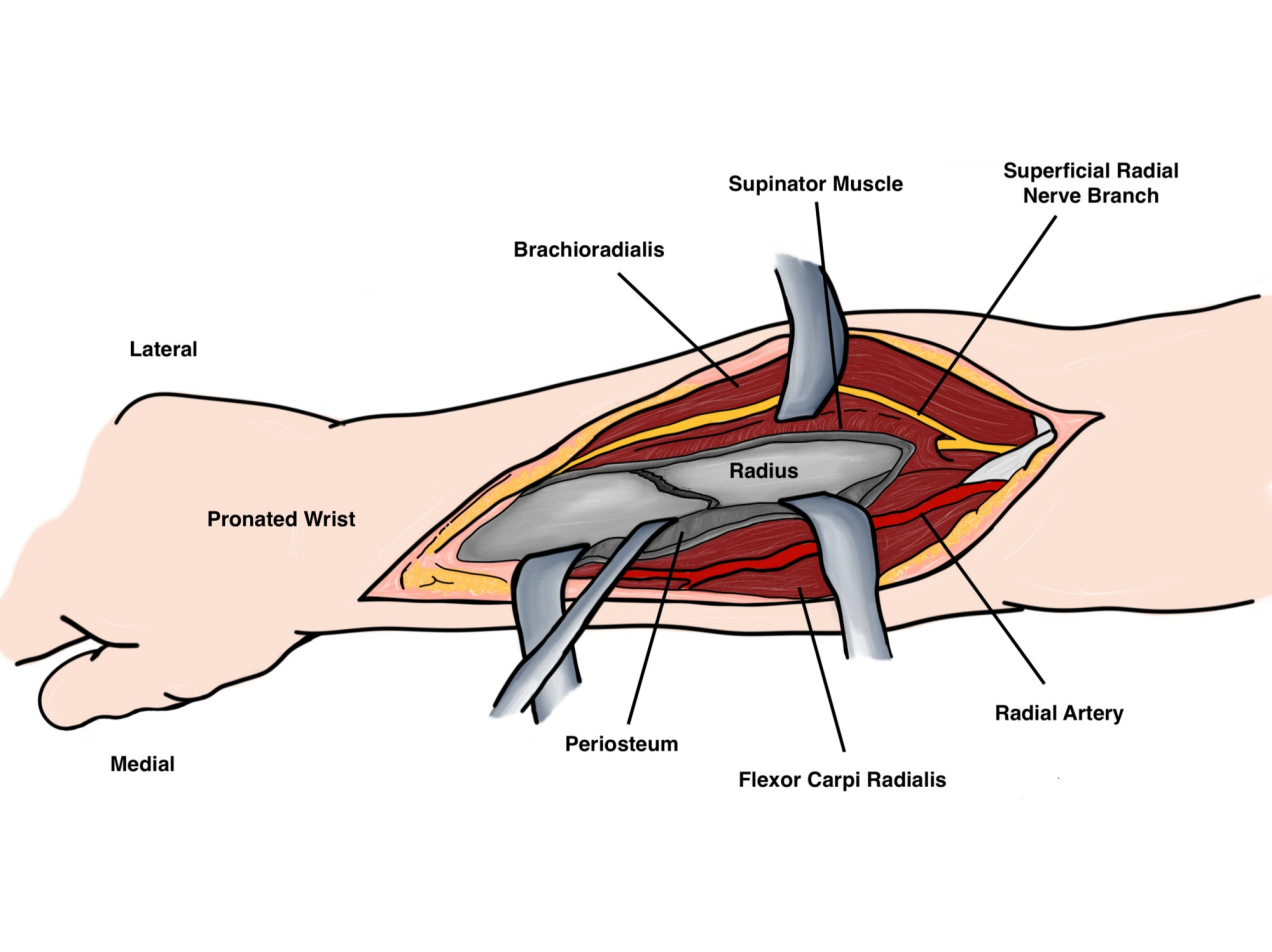
Deep dissection Original illustration by Alswaji G.F.

However, in the middle of the forearm the radius is covered by the flexor digitorum superficialis and the pronator teres (PT). By pronation of the forearm the lateral insertion of the PT on the radius is visible and it may be detached and medially reflected (Figure 11).

Finally in the distal forearm the pronator quadratus and flexor pollicis longus arise from the radius and can be incised laterally with the forearm supinated. Historically, dynamic compression plates have been the preferred method of osteosynthesis in Galeazzi fractures [18]. The efficacy of locked plates has not been extensively studied, but dynamic compression plates have shown to have superior torsional stability than unicortical locked plates [19]. After anatomical reduction of the radius and restoration of the DRUJ, the forearm is examined throughout range of motion and in supination. If the DRUJ is stable and reduced, no further intervention is needed. In cases where the DRUJ is unstable, there are several options; in cases where there is a large ulnar styloid fragment, it can be fixed using lag screw, tension band, or pins. In cases where there is TFCC tear, it can be repaired through a dorsal approach via suture anchors or other techniques [20]. Following repair of the TFCC, or if the surgeon has doubts of the DRUJ stability the DRUJ may be transfixed with K wires transversely with forearm in supination. Finally, the forearm should be mobilized post-operatively in supination to minimize the rotational forces around the DRUJ and to allow for ligamentous healing.

**Figure 12:**
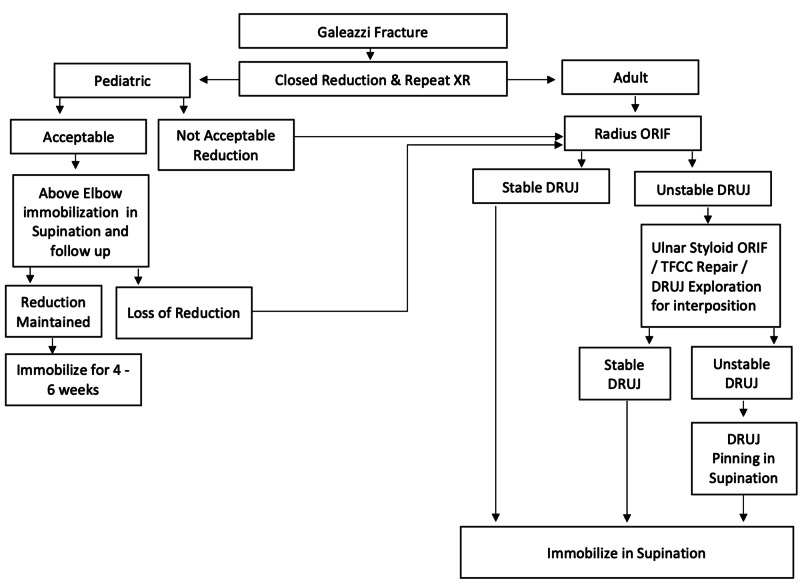
Treatment algorithm Proposed by the author.

On the other hand, in pediatric Galeazzi fractures the gold standard remains non-surgical treatment due to the stable nature of the fracture in this population due to several factors which include: highest elasticity of the ligaments and superior strength of the DRUJ compared to adults, thicker periosteum, and most importantly the higher capacity for the fracture to remodel especially in the joint plane [21]. Also, several studies have reported successful and satisfactory outcomes following non-surgical management of pediatric Galeazzi fractures [2,7,22]. Treatment of pediatric Galeazzi fractures should be in the form of closed reduction under general anesthesia followed by above elbow immobilization in supination for up to six weeks. Immobilization in supinations allows the healing of the TFCC along with maintaining stability of the DRUJ. Although rare, surgical treatment of pediatric Galeazzi is indicated if closed reduction is unable to yield satisfactory alignment or if there is loss of reduction following the initial anatomic reduction. The type of surgical intervention in pediatric may vary depending on the age of the patient, location of the fracture, and stability of the DRUJ after reduction. The options include: K-wire fixation, flexible intramedullary nailing, plate osteosynthesis, or simply open reduction without internal fixation [8,9]. Figure 12 outlines a structured treatment algorithm by the author.

Complications

Along with the usual complications of forearm fractures, the most devastating complications associated with Galeazzi fractures are either radius fracture non-union or malunion, or DRUJ instability which may in turn lead to loss of forearm rotation, reduced grip strength and chronic pain [3,11,12]. For inappropriately treated Galeazzi fractures with late presentation, or with non-reconstructable DRUJ, salvage procedures may be indicated. Salvage procedures include: the Darrach’s procedure, the Sauve-Kapandji procedure, hemiresection or implant arthroplasty. The Darrach’s procedure includes resection of the distal ulna to relive DRUJ pain. Authors vary in describing the amount resected and some preserve the styloid in order to avoid instability [22]. On the other hand, the Sauve-Kapandji procedure is done by arthrodesis of the DRUJ and creating a pseudarthrosis on the ulna just proximal to the site of arthrodesis. The Sauve-Kapandji procedure is superior to Darrach’s procedure as it preserves the ulnocarpal ligaments, and ulnar support of the wrist and it also has a superior aesthetic appearance [23].

## Conclusions

Galeazzi fractures are uncommon type of forearm fractures. They are highly unstable fracture dislocations and they should be addressed in a timely fashion to limit their complications. In adult, the gold standard of treatment is open reduction and internal fixation to overcome the deforming muscular forces and to achieve anatomical reduction of the radial bow and DRUJ. If the reduction of the DRUJ is not achieved on closed manners a trial of open reduction with possible reconstruction of the TFCC or the ulna styloid fracture should be attempted. Pediatric patients, however, can be managed by closed reduction and above elbow immobilization due to their superior bone remodeling capacity and stronger ligaments. Open reduction with or without fixation should be attempted in pediatric patients if close reduction fails to achieve successful results.
